# Do research assessments make college students more reactive to alcohol events?

**DOI:** 10.1186/1940-0640-7-S1-A26

**Published:** 2012-10-09

**Authors:** Molly Magill, Christopher Kahler, Peter Monti, Nancy Barnett

**Affiliations:** 1 Center for Alcohol and Addiction Studies, Brown University, Providence, RI, USA; 2 Department of Behavior and Social Sciences, Brown University, Providence, RI, USA; 3 Department of Community Health, Brown University, Providence, RI, USA; 4 Behavioral and Social Sciences Program in Public Health, Brown University, Providence, RI, USA

## 

Events that precipitate entry into a clinical trial and research procedures may result in short-term gains often seen in control groups in brief motivational intervention (BMI) trials. Such processes may be particularly important among young adults who become engaged in BMI research due to negative alcohol-related events. This study examines effects of alcohol-related events and post-event assessments on changes in college student readiness to change, frequency of alcohol use, and alcohol-related consequences. Students were participants in a longitudinal study of drinking behavior. Participants reporting negative alcohol events were randomized to a post-event assessment (n = 296) or a no-assessment control (n = 196). Those in the post-event assessment condition were interviewed after their event, and participants in both conditions were interviewed three months after their event. Results showed higher readiness to change alcohol use among participants who received a post-event assessment. Across groups, there were significant increases in heavy drinking days prior to the event, and significant reductions post-event, but no post-event differences by assessment group. Moderation analyses showed more post-event reduction in drinking days among assessment group participants with high pre-college alcohol severity. Conversely, participants in the control group with high event aversiveness showed greater reduction in heavy drinking days than those assigned to the assessment group. Findings suggest that college student heavy drinking is reactive to alcohol events, but there were no apparent synergistic effects between an alcohol event and a post-event assessment. Assessment reactivity among students who have experienced an event may depend on alcohol severity and event aversiveness, highlighting the importance of considering possible interactions among extratherapeutic factors in college populations involved in BMI research.

